# Supplementary material for: Mixed methods exploration of Ghanaian women’s domestic work, childcare and effects on their mental health
Source: PLoS One. 2021 Feb 2;16(2):e0245059. doi: 10.1371/journal.pone.0245059 (PMC7853525; doi:10.1371/journal.pone.0245059)

Appendix 1: Thematic network on Understanding the Distribution of Domestic Responsibilities in the Ghanaian Setting and the Changes Over Time

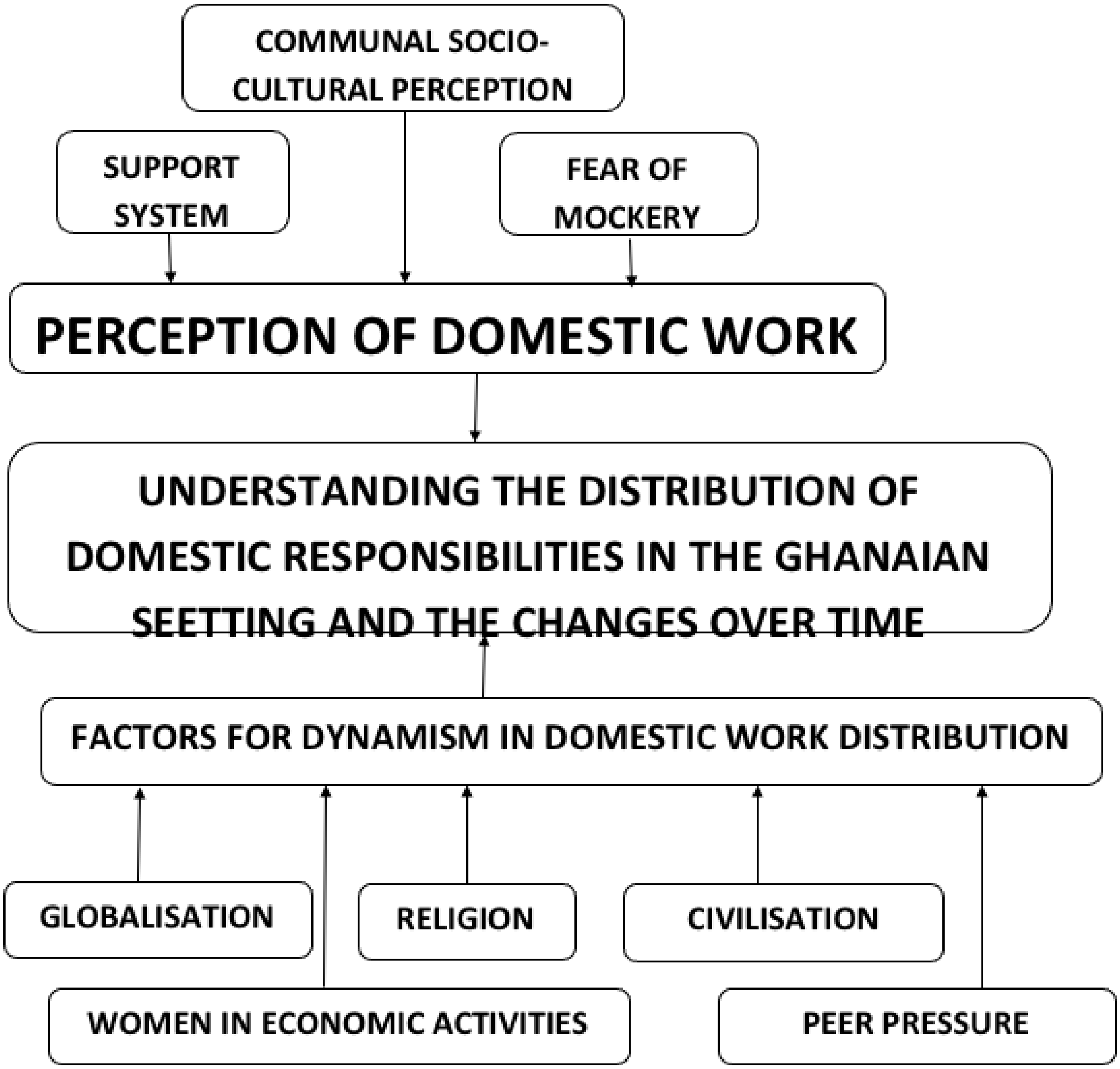

Supplement: S1 File — (PDF) [file pone.0245059.s001.pdf]
