# Supplementary material for: Mixed methods exploration of Ghanaian women’s domestic work, childcare and effects on their mental health
Source: PLoS One. 2021 Feb 2;16(2):e0245059. doi: 10.1371/journal.pone.0245059 (PMC7853525; doi:10.1371/journal.pone.0245059)
Supplement: Attachment — Submitted filename: Mixed method_GHANA.docx [file pone.0245059.s002.docx]

I read the manuscript with great interest and found an interesting analysis of quantitative data. However, the manuscript fails to triangulate both quantitative and qualitative data as it claims to offer. I have the following main observations:

The title needs to be reframed keeping in mind childcare and Ghana

The Introduction fails to highlight the rationale of the study. The need to do this study seems to suggest that because regression estimates would be potentially biased in the past studies and hence this paper offers solution by employing PSM approach. I am not convinced that this study is not adding any value than an analytical refinement.

The authors need to be careful about the use of cut-off classification for mental health. Justification of the cutoff needs to be substantiated with results of other studies in African settings.

Considering the study sampled participants across the five regions, cultural aspects, gender norms, gender roles etc. in these settings need to be discussed in greater detail in light of Women’s Housework and Effects on their Mental Health. Moreover, what is the regional variations and how these regions are different in socio-cultural and economic practices are missing.

The study lacks adequate rational for the choice of methods used to report the study and considerably misses the social-cultural practices across different ethnic groups concerning gender roles, societal view on participation of women in labour force including the current scope and trend of women’s employment outside the household.

The results need to be presented as per the emerging themes in the qualitative part of the study. While the quantitative method is elaborated somewhat descriptively but there is no adequate information about qualitative data collection process and analysis including triangulation of both data that fails to justify the design used to study the research problem.

The qualitative data is presented without any background and discussion after the quantitative data is reported. Ideally, while triangulating, both findings (quantitative and qualitative) must flow seamlessly. However, this study reports findings from quantitative and qualitative separately that fails to address the purpose of mixed method research.

The findings need to be discussed in greater details which seems completely a miss now. The inference drawn in conclusion, “more efforts should be made to lessen the incompatibilities between these dual roles. This can be done through institutional factors like the provision of childcare services.” seem out of context. Similar inferences must not be drawn.
