# Supplementary material for: Mixed methods exploration of Ghanaian women’s domestic work, childcare and effects on their mental health
Source: PLoS One. 2021 Feb 2;16(2):e0245059. doi: 10.1371/journal.pone.0245059 (PMC7853525; doi:10.1371/journal.pone.0245059)
Supplement: Attachment — Submitted filename: Response to Reviewer Comments_2 _N.docx [file pone.0245059.s004.docx]

**Comments from academic editor:**

The reviewers still feel some minor revisions are required before recommending this paper for publication. Alongside the reviewer suggestions, I request authors to go for a professional English proof reading of the paper and also make the stylistic changes according to PLOS One author guidelines.

**Response:**

We are grateful for the reviewers’ comments and have made the suggested changes, as requested. We have also submitted the paper for proof reading and have adhered to the journal’s format.

**Reviewer #1**

The authors have addressed all the comments and revised version of the manuscript is up to the level of satisfaction. There are a few minor suggestions the authors may please consider.

**Comment:**

In page no 7, the authors reported "snowballing sampling" as one of the sample selection procedures. It is suggested that the authors report it as "Snowball Sampling".

**Response:**

This change has been made-thank you.

**Comment:**

As per the information provided in paragraph 3 page number 8, it is unclear if the researchers used inductive approach to develop qualitative codes or deductive approach to do so. It would be better if authors could add a sentence or two clarifying it.

**Response:**

An inductive approach was used, and this has been clarified in the text.

**Comment:**

There is inconsistency in table numbers and the reporting of tables in the text. For example, table 1 comes two times (the first and second tables are numbered as table 1). Table 2 reported in paragraph 1 of descriptive results section (page 11) is not found. The authors may please check this.

**Response:**

Thank you; this inconsistency in the table labels has been addressed.

**Comment:**

The researchers may please check that the referencing is consistent and adheres to the standard format. For example; DOI was not reported in few references though DOI was available. Similarly, some references such as Teychenne at al. (2008) needs to be formatted.

**Response:**

Thank you for these comments- these changes have been made.

**Reviewer #2**

Most of the comments are addressed and the current version of manuscript appears to have improved. However, there still are a few minor concerns especially, the language used in the manuscript needs substantial revision. Additionally, the discussion and conclusion section need a major revision.

**Comment:**

The choice of word/phrase such as ‘sexual division of labour’ in page 3, ‘spending four times more effort’ in page 3, ‘wives’, ‘in order to end up with valid and well-substantiated conclusions’ in page 5, ‘five-level response’ in page 6 etc. to indicate a few, needs definite revision. Language consistency may be maintained.

**Response:**

“Sexual division of labor” revised to “gendered division of labour”

“spending four times more effort” revised to “spend 4 times as much time”

“in order to end up with valid and well-substantiated conclusions” revised to “in order to draw valid conclusions”

“five-level response” maintained to indicate that each question on respondent’s emotional state expected to prompt one of 5 responses.

**Comment:**

Considering the current version of the manuscript is rather long, it would be useful to use sub-heads throughout the manuscript.

**Response:**

This comment is well received, and this is what we sought to do with the last revision that was submitted:

The current structure is as follows:

I. Introduction
II. Data and Methodology
    a. Quantitative Data
       i. Selected variables of interest
    b. Qualitative Data
    c. Methods
III. Results
     a. Descriptive results
     b. Results from regressions and qualitative data
        i. Overburdened
        ii. Physical effects
        iii. Economic effects
     c. Robustness checks for selection and reverse causality
IV. Discussion of results
V. Conclusion

We are happy to revise the current structure further as per the reviewer’s suggestions.

**Comment:**

At certain places in the introduction, the authors have expressed their views, for instance in page 4 ‘Therefore, when women’s total domestic workload is high, the combination of different roles may actually damage her health. Furthermore, the influence of social economic status may moderate the relationship. On the one hand, women with higher socioeconomic status may experience lower mental distress if they are better able to afford domestic appliances and house helps to reduce time spent in domestic work. On the other hand, wealthier women who spend more time in domestic work may have poorer mental health outcomes if this increases the opportunity costs of earnings and/or leisure activities, among other reasons’. Such blanket statements can lose the focus of the argument. The authors need to use appropriate scientific reporting style.

**Response:**

Thank you for this comment. To clarify, the use of the word “may” is meant to present the authors’ hypotheses without asserting a firm stance before the scientific analyses are completed. Additionally, the presentation of two potentially dissenting effects of social status on mental health is meant to, again, to objectively explain that no conclusions can be drawn until these relationships are tested, as either outcome is possible.

**Comment:**
Use of italics may be avoided unless essential

**Response:**

Thank you- as per the reviewer’s comment, all italicized text has been revised.

**Comment:**

Referencing style needs to be consistent for eg. In page 5 (Adjei and Brand, 2018; Everard et al., 2000), Lawlor et al. (2002) is inappropriate.

**Response:**

Thank you- the referencing style has been checked for consistency.

**Comment:**

The manuscript is lengthy and commentary such as ‘The remainder of the paper is scheduled as follows: Section II describes the (qualitative and quantitative) data and empirical methodologies employed in this research. Section III presents results and discussions of research findings, while Section IV concludes with potential social and policy implications of this research’ in page 5 may be avoided.

**Response:**

Thank you- this section has been removed.

**Comment:**

Details of data availability, funding and other support in the survey may be referred to the report if available or be placed in the supplementary file.

**Response:**

While we had previously refrained from including this information in earlier versions of the paper, reviewer(s) disagreed. We are happy to defer to a universally agreed-upon position by the reviewers and editor in keeping or eliminating this from the main document.

**Comment:**

Policy implications may be written in light of the findings and requires to be revised to tone down the ambitious claims made.

**Response:**

The policy applications of the research, as stated in the conclusion, include:

1. Efforts should be made to encourage greater participation of men in domestic work. One way to do this is with early socialization of boys and girls
2. Importance of labour saving devices to reduce domestic work burdens that women face and subsequently, improve their mental health.

These prescriptions are not necessarily ambitious. We already find, from our qualitative surveys, that men are gradually willing to assist with domestic work, particularly childcare, where in the past, it was considered unthinkable. These recommendations are meant to encourage such behaviors and prescribe early socialization of *both* boys and girls as one way to achieve this.

We would like to highlight that the following prescription has been removed from the abstract “Additionally, family-friendly polices to reduce work-family incompatibilities that women face should be promoted. These may include publicly provided daycare centers and even nursing stations and childcare centers within workplaces.”

**Comment:**
Shouldn’t the results and discussion sections be separate?

**Response:**

Thank you- they are separate, and the headings have been revised to reflect this

**Comment:**
Although the manuscript has improved from the previous version but would need a careful copy edit before publication.

**Response:**

Thank you, this has been done.

**Reviewer #3:**

Overall performance of this manuscript is quiet interesting and suitable for the journal like 'PLOSE ONE'

**Response:**

Thank you.
