# Supplementary material for: Mixed methods exploration of Ghanaian women’s domestic work, childcare and effects on their mental health
Source: PLoS One. 2021 Feb 2;16(2):e0245059. doi: 10.1371/journal.pone.0245059 (PMC7853525; doi:10.1371/journal.pone.0245059)
Supplement: Attachment — Submitted filename: Response to Reviewer Comments.docx [file pone.0245059.s003.docx]

*Working Papers Folder: Childcare Distribution and Welfare Outcomes*

**Journal Requirement:**

**Comment:**

When submitting your revision, we need you to address these additional requirements.

1. Please ensure that your manuscript meets PLOS ONE's style requirements, including those for file naming. The PLOS ONE style templates can be found at

<https://journals.plos.org/plosone/s/file?id=wjVg/PLOSOne_formatting_sample_main_body.pdf> and

<https://journals.plos.org/plosone/s/file?id=ba62/PLOSOne_formatting_sample_title_authors_affiliations.pdf>

**Response:** The manuscript has been modified in accordance with the PLOSE ONE style as indicated in the reference documents provided

**Comment:**

Thank you for your ethics statement: "Name of Institutional Board: University of Ghana Ethics Committee for the Humanities (ECH)

Approval Number: ECH 127/18-19

Consent: It was written. It was read out to participants of the study and they appended signatures(or thumb print) to indicate consent."

i) Please amend your current ethics statement to confirm that your named institutional review board or ethics committee specifically approved this study.

ii) Once you have amended this/these statement(s) in the Methods section of the manuscript, please add the same text to the “Ethics Statement” field of the submission form (via “Edit Submission”).

For additional information about PLOS ONE ethical requirements for human subjects research, please refer to <http://journals.plos.org/plosone/s/submission-guidelines#loc-human-subjects-research>.

**Response:** This has been done in the online submission portal

**Comment:**

We note that you have indicated that data from this study are available upon request. PLOS only allows data to be available upon request if there are legal or ethical restrictions on sharing data publicly. For information on unacceptable data access restrictions, please see <http://journals.plos.org/plosone/s/data-availability#loc-unacceptable-data-access-restrictions>.

In your revised cover letter, please address the following prompts:

a) If there are ethical or legal restrictions on sharing a de-identified data set, please explain them in detail (e.g., data contain potentially identifying or sensitive patient information) and who has imposed them (e.g., an ethics committee). Please also provide contact information for a data access committee, ethics committee, or other institutional body to which data requests may be sent.

b) If there are no restrictions, please upload the minimal anonymized data set necessary to replicate your study findings as either Supporting Information files or to a stable, public repository and provide us with the relevant URLs, DOIs, or accession numbers. Please see <http://www.bmj.com/content/340/bmj.c181.long> for guidelines on how to de-identify and prepare clinical data for publication. For a list of acceptable repositories, please see <http://journals.plos.org/plosone/s/data-availability#loc-recommended-repositories>.

We will update your Data Availability statement on your behalf to reflect the information you provide.

**Response:** Although we are happy to share the qualitative data associated with the study, the terms of the contract with the funders does not allow the researchers to make the data publicly available before the termination of the project. The data sharing arrangements per the contract with the funders is indicated as follows: “*We agree to make the data publicly available as soon as possible but no later than within one year of the completion of the funded project period for the parent award or upon acceptance of the paper for publication, whichever is earlier”*. Per this provision, we are unable to make the data publicly available since the project has not yet ended.

4. Please ensure that you refer to Figure 1 in your text as, if accepted, production will need this reference to link the reader to the figure.

**Response:**

This has been done.

**Comment:**

5. ** Please include your tables as part of your main manuscript and remove the individual files **. Please note that supplementary tables (should remain/ be uploaded) as separate "supporting information" files

**Response:** This has been done. Thank you.

**Additional Editor Comments (if provided):**

All three reviewers find there is merit in your manuscript. However, they come up with several suggestions which I felt are very important. If your willing to revise and resubmit the paper according to reviewer comments, we are willing to consider this. The manuscript fails to triangulate both quantitative and qualitative data as it claims to offer. Please take this suggestion and revise the paper carefully. However, this doesn't mean the revised version is automatically accepted. It will be re-evaluated by me and the reviewers if needed.

**Response:**
We are grateful to the reviewers for the comments provided and have given serious considerations to each suggestion. The manuscript has been revised accordingly. We would however like to state that in more than one case, we experienced some difficulty in understanding what changes were being suggested by Reviewer 3.

**Reviewer 1**

**Comment:**

The research paper is well written and adds to an important domain. The use of mixed methods approach, use of GESPS data and sound use of statistical tools are the strengths of the paper. However, there are some important areas which the authors may consider to look at.

**Response:**

Thank you.

**Comment:**
Though the authors provided a great detail of information about GSEPS, it is important that they inform how they accessed the data, process involved and acknowledge the source of the data. It is essential since the providers of the secondary data require them to be acknowledged in the manuscripts published using the data.

**Response:**

The following information is provided in the section describing the Quantitative data (i.e. Section IIa):

The Ghana Socioeconomic Panel Survey is a joint effort between the Economic Growth Centre (EGC) at Yale University and the Institute of Statistical, Social and Economic Research (ISSER), at the University of Ghana (Legon, Ghana). The survey is principally funded by the EGC, designed by both the EGC and ISSER, and carried out and supervised by ISSER. Technical support is provided by the University of Michigan’s Survey Research Center International Unit (SRC IU). Although wave 1 is publicly available, (<https://microdata.worldbank.org/index.php/catalog/2534>), Wave 2 data is available only upon request. Interested researchers are asked to provide information about themselves and the use to which they will put the data before access is granted.

**Comment:**
The researchers reported the use of a total of 60 qualitative interviews (50 couples and 10 key informants). However, a limited information was provided regarding measures taken to ensure the rigor of the qualitative data collected. The researchers may please provide the detailed outline of the same. It is also advised to provide a table outlining the characteristics of the Interview participants, it will establish that participants are best fit with regard to this study.

**Response:**

Additional information has been provided on measures taken to ensure the rigor of the qualitative data collected. Additionally, a table, with some discussion, has been provided that describes the characteristics of the interviewed couples.

**Comment:**

The researchers need to provide a detailed outline on the data collection (if recording was used or not, if yes if consent obtained, language of the interview, validation of the interview by the respondents), and data analysis procedures followed to collect and analyze qualitative data.

**Response:**

This has been provided in Section IIb.

**Reviewer 2**

**Comment:**

I read the manuscript with great interest and found an interesting analysis of quantitative data. However, the manuscript fails to triangulate both quantitative and qualitative data as it claims to offer. I have the following main observations:

**Comment:**

The title needs to be reframed keeping in mind childcare and Ghana

**Response:**

The current title is *Mixed Methods Exploration of Women’s Housework and Effects on their Mental Health*. Following the reviewer’s suggestion, we are happy to amend this to the following title: *Mixed Methods Exploration of Ghanaian Women’s Domestic Work, Childcare and Effects on their Mental Health*

**Comment:**

The Introduction fails to highlight the rationale of the study. The need to do this study seems to suggest that because regression estimates would be potentially biased in the past studies and hence this paper offers solution by employing PSM approach. I am not convinced that this study is not adding any value than an analytical refinement.

**Response:**

Many thanks for this observation. I have made the motivating arguments on potential mental health implications of domestic burden more explicit, particularly in the present context.

**Comment:**

The authors need to be careful about the use of cut-off classification for mental health. Justification of the cutoff needs to be substantiated with results of other studies in African settings.

**Response:**

Many thanks for this comment. We have provided references to indicate that these cut-offs have been adopted in recent studies on both Africa in general, an on Ghana, in particular.

**Comment:**

Considering the study sampled participants across the five regions, cultural aspects, gender norms, gender roles etc. in these settings need to be discussed in greater detail in light of Women’s Housework and Effects on their Mental Health. Moreover, what is the regional variations and how these regions are different in socio-cultural and economic practices are missing. The study lacks adequate rational for the choice of methods used to report the study and considerably misses the social-cultural practices across different ethnic groups concerning gender roles, societal view on participation of women in labour force including the current scope and trend of women’s employment outside the household.

**Response:**

A discussion on differences in sociocultural norms and economic practices between women from northern and southern Ghana has been included.

**Comment:**

The results need to be presented as per the emerging themes in the qualitative part of the study. While the quantitative method is elaborated somewhat descriptively but there is no adequate information about qualitative data collection process and analysis including triangulation of both data that fails to justify the design used to study the research problem.

**Response:**

Thank you for this comment. We have discussed the themes more fully in the revised document. We have also provided greater information about the data collection process and triangulated both quantitative and qualitative data more effectively.

**Comment:**

The qualitative data is presented without any background and discussion after the quantitative data is reported. Ideally, while triangulating, both findings (quantitative and qualitative) must flow seamlessly. However, this study reports findings from quantitative and qualitative separately that fails to address the purpose of mixed method research.

**Response:**

Thank you for these helpful comments. We have attempted to integrate the findings from the qualitative surveys more comprehensively into quantitative results, rather than reporting results from each analysis separately.

**Comments:**

The findings need to be discussed in greater details which seems completely a miss now. The inference drawn in conclusion, “more efforts should be made to lessen the incompatibilities between these dual roles. This can be done through institutional factors like the provision of childcare services.” seem out of context. Similar inferences must not be drawn.

**Response:**

These concerns are well-noted. We have limited our recommendations to more direct findings from the research.

**Reviewer 3**

**Comment:**

The abstract need to be more scientific, structured with summary of key findings.

**Response:**

Thank you. We are however not aware that this journal requires/requests abstracts to be submitted in a structured format. The reviewer may kindly confirm via the following link: <https://journals.plos.org/plosone/s/file?id=wjVg/PLOSOne_formatting_sample_main_body.pdf>

**Comment:**
The introduction is too extensive and superfluous. It should not be above 850 words.

**Response:**

Thank you for your comment. The journal does not appear to have a word limit for the introduction. It is a little lengthy because in providing the motivation for the study, we have attempted to indicate the gap that we are filling by providing a brief review of the existing literature. We have also introduced a couple of theories that we believe provide a much-needed underpinning for this work.

**Comment:**
The content is not written in scientific. The objectives of this work need to be more focused into the analysis.

**Response:**

We apologize that we do not quite understand the comments here and what changes are being suggested by the reviewer.

**Comment:**
Result and discussion should be following independently.

**Response:**

Thank you for this. We have included a discussion section, as requested by the reviewer.

**Comment:**
The regression and multinomial logistic model didn’t explained proper scientific way and incomplete to proof the goals of the paper. Results are undependable as statistical results are merely stated without any analytical explanation.

**Response:**

We apologize that we do not quite understand the comments here and what changes are being suggested by the reviewer.

**Comment:**
Quantitative Analysis should be more in-depth, scientific and context specific for the specific area. And study is need for more information on policy implications of these particular findings.

**Response:**

Thank you. We have updated the conclusion section with policy recommendation derived more directly from the research findings.

**Comment:**
It uses inappropriate statistical tools for Table 3 & 4.

**Response:**

We apologize that we do not quite understand the comments here and what changes are being suggested by the reviewer.
